# Mendelian Randomization Analysis of the Association of SOCS3 Methylation with Abdominal Obesity

**DOI:** 10.3390/nu14183824

**Published:** 2022-09-16

**Authors:** Yuqian Li, Xiaotian Liu, Runqi Tu, Jian Hou, Guihua Zhuang

**Affiliations:** 1 Departmentof Epidemiology and Biostatistics, School of Public Health, Xi’an Jiaotong University Health Science Centre, Xi’an 710061, China; 2 Department of Clinical Pharmacology, School of Pharmaceutical Science, Zhengzhou University, Zhengzhou 450052, China; 3 Department of Epidemiology and Biostatistics, School of Public Health, Zhengzhou University, Zhengzhou 450052, China

**Keywords:** abdominal obesity, SOCS3, methylation, single-nucleotide polymorphism, Mendelian randomization

## Abstract

This study was conducted to evaluate the potential causality association of SOCS3 methylation with abdominal obesity using Mendelian randomization. A case–control study, including 1064 participants, was carried out on Chinese subjects aged 18 to 79. MethylTargetTM was used to detect the methylation level for each CpG site of SOCS3, and SNPscan^®^ was applied to measure the single-nucleotide polymorphism (SNP) genotyping. The logistic regression was used to assess the relationship of SOCS3 methylation level and SNP genotyping with abdominal obesity. Three types of Mendelian randomization methods were implemented to examine the potential causality between SOCS3 methylation and obesity based on the SNP of SOCS3 as instrumental variables. SOCS3 methylation levels were inversely associated with abdominal obesity in five CpG sites (effect estimates ranged from 0.786 (Chr17:76356054) to 0.851 (Chr17:76356084)), and demonstrated positively association in 18 CpG sites (effect estimates ranged from 1.243 (Chr17:76354990) to 1.325 (Chr17:76355061)). The causal relationship between SOCS3 methylation and abdominal obesity was found using the maximum-likelihood method and Mendelian randomization method of penalized inverse variance weighted (MR-IVW), and the *β* values (95% *CI*) were 5.342 (0.215, 10.469) and 4.911 (0.259, 9.564), respectively. The causality was found between the SOCS3 methylation level and abdominal obesity in the Chinese population.

## 1. Introduction

Obesity, a chronic metabolic disease, has attracted increasing attention worldwide [1]. Especially abdominal obesity, characterized by abnormal accumulation of fat in the abdomen, is associated with increased prevalence of numerous chronic non-communicable diseases, such as diabetes and cardiovascular diseases [2]. Epidemiological evidence indicated that the global prevalence of obesity has increased rapidly, from 3% in 1975 to 11% in 2016 among men, and correspondingly from 6% to 15% among women [3]. Moreover, previous research showed that the abdominal obesity prevalence has exceeded 30% in the Spanish population (31.2% in the subjects aged 3 to 24 years and 33.4% in the adults aged 25 to 64 years, respectively) [4,5]. In addition, a cross-sectional study, including 441,306 participants, showed that the abdominal obesity prevalence was 29.1% (277.8 million) among Chinese adults [6]. Therefore, it is necessary to explore the mechanisms of the genes and epigenetics in abdominal obesity development to prevent and control abdominal obesity and its complications, as well as reduce the disease burden.

The development of abdominal obesity is closely related to acquiring behavioral factors. However, genetic and epigenetic factors are the prerequisite for the occurrence of obesity status, which determines whether an individual is at high risk of obesity. Previous evidence suggested that the suppressor of cytokine signaling 3 (SOCS3) played an essential role in the regulation of energy metabolism homeostasis, which inhibited the activity of insulin and leptin (characteristic feature in human obesity) [7,8]. One study in the Northern European population pointed out that the association of SOCS3 methylation level with obesity was significantly inverse [9]. A study conducted in a community population in New Haven revealed that subjects with obesity had lower methylation levels of SOCS3 [10]. In addition, an epigenome-wide study in 1066 women from the Sister Study cohort revealed that the SOCS3 methylation level decreased by 0.05% with each one-unit increase in body mass index (BMI) [11]. The above research highlighted the necessity to explore the substantial role of SOCS3 methylation under abnormal metabolic conditions, such as abdominal obesity.

Waist circumference (WC) is a crucial driver of risk for cardiovascular disease [12], and evidence from Chinese adults found that the association of type 2 diabetes risk with changes in WC was more robust than that with changes in BMI [13]. In addition, a previous study showed that obesity-related health risk was explained by WC and not by BMI in 14,924 adult participants who took part in the Third National Health and Nutrition Examination Survey [14], and the association of WC with T2DM was independent of BMI [13]. To date, studies involved in the association of SOCS3 methylation with abdominal obesity have been limited, especially those conducted to infer the etiology of this association in an observational study. The purpose of this study was to evaluate the association between SOCS3 methylation and abdominal obesity. Furthermore, we conducted Mendelian randomization studies to explore whether there was causality between SOCS3 methylation levels and abdominal obesity by using the SNP of SOCS3 as instrumental variables.

## 2. Materials and Methods

### 2.1. Study Population

The participants came from the Henan Rural Cohort Study, which was conducted from July 2015 to September 2017 in five countries of Henan province in central China, using a multi-stage cluster sampling method. The respondents in this cohort were local residents aged from 18 to 79 years old and had no serious psychosomatic diseases. Detailed information for the cohort study has been elaborated on elsewhere [15]. A total of 1067 participants were chosen from the baseline survey, and 1064 participants were included in final analyses, after excluding 3 subjects who had no methylation information. The current study was approved by the Life Science Ethics Committee of Zhengzhou University, and informed consent was signed by every subject before the survey began.

### 2.2. Data Collection and Laboratory Measurements

The basic information of the subjects was collected via in-person interviews, and the physical examination was conducted in strict accordance with the operating manual by trained investigators. Demographic characteristics included age, gender, education level (elementary school or below, junior high school or above), marital status (married/cohabiting, widowed/single/divorced/separation), and average monthly family income (CNY < 500, CNY 500~, and CNY ≥ 1000). Behavioral risk factors included smoking status (never, ever, and current smoking), drinking status (never, ever, and current drinking), and physical activity (divided into low, medium, and high groups according to the International Physical Activity Questionnaire); dietary habits included high-fat diet (livestock and poultry meat ≥ 75 g/day) and more vegetables and fruits intake (≥500 g/day). In addition, family history of diabetes was defined as having at least one immediate family member who ever had or was currently suffering from diabetes.

After fasting for over 8 h, 5.0 mL of non-anticoagulant venous blood and 10.0 mL of anticoagulant venous blood (EDTA-K_2_) were collected from the subjects. The whole blood was isolated from anticoagulant blood and was used to extract DNA.

### 2.3. Abdominal Obesity Assessment

Individuals with a WC ≥ 90 cm in men or ≥80 cm in women were defined as those with abdominal obesity, excluding pregnant women [16]. The non-abdominal obese group was defined as a WC < 90 cm in men and <80 cm in women. When measuring the WC, the subjects were asked to stand upright, with their abdomen relaxed, and normal breathing. The measuring tape was close to the skin at 1.0 cm above their navel while wearing light clothes. The reading was accurate to 0.1 cm. The WC was measured twice, and the mean was taken as the final measurement value.

### 2.4. DNA Methylation and Genotyping of SOCS3

DNA of the participant was isolated by using the Whole Blood Genomic DNA Medium extraction kit III (Bioteke Corporation, Beijing, China). Detection of the DNA methylation level of SOCS3 was accomplished by MethylTarget™ (Genesky Corporation, Shanghai, China). According to the previous study [17], the CpG site of the SOCS3 gene was chosen according to the following standards: (1) observed/expected ratio > 0.60; (2) percent C and G > 50.00%; (3) length > 200 bp. In this study, the methylation levels of four CpG regions (Chr17:76355136–76355350, Chr17:76356054-76356232, Chr17:76354927-76355115, and Chr17:76354582–76354763, which included 93 CpG sites) were detected. The methylation level of each CpG site was defined as the proportion of the methylated cytosines that accounted for the total tested cytosines [18]. Each tested CpG site was named as its genomic position. Genomic position can be described as the chromosomal location of each CpG site according to the assembly GRCh37/hg19.

The genotypes of SOCS3 were detected by the SNPscan™ Kit (Genesky Corporation, Shanghai, China). A total of six SNPs in SOCS3 were included in the study based on domestic and international references, combined with bioinformatics databases and HapMap databases (according to the criteria of R^2^ > 0.8 in linkage disequilibrium analyses and minimum allele frequency > 0.05 of SNP).

### 2.5. Statistical Analysis

Continuous variables were shown as mean ± standard deviation (SD), while the SOCS3 methylation level was expressed as medium ± interquartile range (IQR), and the differences between the abdominal obese group and the non-abdominal obese group were compared by Student’s *t*-test. Categorical variables were represented by frequency (percentage), and the differences between the two groups were compared using Pearson’s chi-square test. The Hardy–Weinberg equilibrium (HWE) test was conducted for each SNP using a chi-square test among the controls. The logistic regression model was fitted to assess the effect estimates (odds ratio (OR) and 95% confidence interval (CI)) per IQR increase in SOCS3 methylation level on abdominal obesity. The adjusted potential confounding factors included age, gender, education levels, marital status, average monthly family income, smoking status, drinking status, physical activity, high-fat diet, and more vegetable and fruit intake. Moreover, the restricted cubic spline analysis was used to inquire into the non-linear dose-response relationship between SOCS3 methylation levels and abdominal obesity (knots located at the 25th, 50th and 75th percentiles of the SOCS3 methylation levels). In addition, the chi-square test was used to test the Hardy–Weinberg equilibrium in the control group at each SNP to detect whether the population was representative [19]. The associations of SNP with abdominal obesity were evaluated by logistic regression models. Associations between SNP and methylation levels of SOCS3 were examined by univariate regression analyses. Furthermore, three Mendelian randomization methods, including maximum-likelihood method, Mendelian randomization method of penalized weighted median (MR-median) and Mendelian randomization method of penalized inverse variance weighted (MR-IVW), were used to explore the causal association between SOCS3 methylation levels and abdominal obesity. There were three assumptions of Mendelian randomization analysis, which were as follows: 1. the instrumental variable should be robustly associated with the exposure; 2. the instrumental variable should not be associated with confounding factors of the exposure-outcome association; 3. the instrumental variable should affect outcome merely through exposure, not via alternative pathways [20]. The latter two assumptions were called no horizontal pleiotropy of the instrumental variable, which could be detected by the Mendelian randomization of the Egger (MR-Egger) regression intercept [21]. Moreover, the Wald ratio was used to estimate the causal effect of DNA methylation (exposure, x) at SNP-related CpG sites on abdominal obesity (outcome, y) and the change in abdominal obesity per standard deviation (SD) increase in methylation, using the formula *β*Y|G^/*β*X|G^ for each instrumental SNP (G), where *β*Y|G^ is the SD change in abdominal obesity per copy of the effect allele of each SNP and where *β*X|G^ is the SD increase in methylation per copy of the effect allele of each SNP [22,23].

All statistical analyses were performed using the SAS 9.1 software package (SAS Institute, Cary, NC, USA) and R software (version 3.6.1, R Development Core Team, Vienna, Austria), and *p* < 0.05 at two tails was considered statistically.

## 3. Results

### 3.1. Characteristics of Participants

Table 1 shows the basic characteristics of all subjects (471 subjects with and 593 subjects without abdominal obesity). The mean age (mean ± SD) was 58.70 ± 8.46 years and 60.17 ± 8.78 years in participants with abdominal obesity and without abdominal obesity, respectively (*p* < 0.05). In the abdominal obese population, women (70.7%) and those with moderate physical activity (48.0) accounted for a higher proportion. In addition, individuals with abdominal obesity tended to be non-smokers (80.5%). There were no statistical differences in the distribution of other variables between the abdominal obese group and the non-abdominal obese group.

### 3.2. Association of SOCS3 Methylation Levels with Abdominal Obesity

Methylation levels of 93 CpG sites of the SOCS3 gene were measured and evaluated. Table 2 represents the significant association between the methylation levels of 23 CpG sites and abdominal obesity (all *p* < 0.05), and Table S1 describes no statistical association of the other 70 CpG sites with abdominal obesity in the adjusted models. Of the 23 statistically significant methylation sites, the methylation levels of 5 CpG sites were inversely associated with abdominal obesity (Chr17:76356054, Chr17:76356084, Chr17:76356099, Chr17:76356178 and Chr17:76356190), and effect estimates (*OR* (95% *CI*)) were 0.786 (0.672, 0.919), 0.851 (0.732, 0.988), 0.838 (0.719, 0.976), 0.795 (0.679, 0.930) and 0.841 (0.721, 0.983), respectively. The methylation levels of the remaining 18 CpG sites in one CpG regions (Chr17:76354927-76355115) were positively associated with abdominal obesity, and effect estimates ranged from 1.243 (Chr17:76354990) to 1.325 (Chr17:76355061).

Table S2 and Figure S1 indicate the results of restrictive cubic spline analyses between 23 CpG sites and abdominal obesity. Except for the Chr17:76356054 site (*p* for the non-linear association test was 0.025), the dose–response relationships were linear between SOCS3 methylation levels (the remaining22 CpG sites) and abdominal obesity in the adjusted models (all *p* for the non-linear association tests were >0.05).

### 3.3. Effect Estimates between SNP of SOCS3 and Abdominal Obesity

Table 3 summarizes the associations between SNP of SOCS3 and abdominal obesity. The six SNPs were in accordance with the Hardy–Weinberg equilibrium (all *p* > 0.05). Rs9914220 was associated with abdominal obesity with each mutant allele increase, and the *OR* (95% *CI*) was 0.823 (0.686, 0.988). No significant associations were found between the other five SNPs of SOCS3 and abdominal obesity (all *p* > 0.05).

### 3.4. Causal Estimates of SOCS3 Methylation Level on Abdominal Obesity

The associations between the six SNPs and methylation levels of twenty-three CpG sites of SOCS3 were explored. Significant associations were only found between five SNPs (rs12953258, rs4969168, rs2280148, rs4969170, rs9914220) and methylation levels of twenty CpG sites of SOCS3 (Table S3). Combined with the results of Table 3, only four SNPs (rs12953258, rs4969168, rs2280148, rs4969170) and associated seventeen CpG sites (Chr17:76356084, Chr17:76356099, Chr17:76354963, Chr17:76354984, Chr17:76354990, Chr17:76355044, Chr17:76355061, Chr17:76355068, Chr17:76355089, Chr17:76355115, Chr17:76354927, Chr17:76354955, Chr17:76354965, Chr17:76355009, Chr17:76355014, Chr17:76355017 and Chr17:76355029) were included into the Mendelian randomization analyses. The MR-Egger intercept test indicated no horizontal pleiotropy (*p* = 0.726).

Table 4 indicates the results of the Mendelian randomization analyses by three methods for the causal estimates of SOCS3 methylation level on abdominal obesity. The effect estimates (*β* (95% *CI*)) on abdominal obesity were 5.342 (0.215, 10.469) and 4.911 (0.259, 9.564) using the maximum-likelihood method and MR-IVW method, respectively, while the corresponding effect was 5.117 (−1.356, 11.590) using the MR-median method. In addition, the Wald ratio of each CpG site is shown in the Table S5.

## 4. Discussion

The current research demonstrated that 5 CpG sites methylation levels of SOCS3 were inversely associated with abdominal obesity, and methylation levels of 18 CpG sites were positively associated with abdominal obesity. The dose–response relationships between SOCS3 methylation levels and abdominal obesity were linear. Furthermore, the Mendelian-randomization analyses indicated a causal association between SOCS3 methylation levels and abdominal obesity.

The evidence emphasized that DNA methylation within intergenic regions plays a potential role in inhibiting the genetic expression, and those occurring in CpG islands might stably silence gene expression [24]. An accumulating body of research has shown that DNA methylation was closely related to aberrant glucose metabolism and dysregulation lipid metabolism, which represents a central role in metabolic and cardiovascular disease [25,26,27]. In addition, previous studies have indicated that the SOCS3 methylation level was negatively linked to type 2 diabetes [17,28,29]. A case–control study in young women summarized that the methylation levels of cg18181703 in SOCS3 (chr17: 76354621) significantly differed between obesity and lean individuals (−0.245 (−0.332, −0.142), *p* <  0.05) [30]. Another study conducted on community volunteers (aged 18–50) also found that the methylation level of cg18181703 of SOCS3 was lower in the obese subjects (3.08 (2.04, 4.71)) [10]. Moreover, an epigenome-wide association study in an Arab population revealed that the methylation level of cg18181703 of SOCS3 was negatively associated with BMI [31].

Previous studies have focused on the relationship between the methylation level of cg18181703 SOCS3 and obesity. However, the reports on exploring the associations between SOCS3 methylation levels and abdominal obesity (defined by WC) are limited. The current study indicated significant differences in SOCS3 methylation levels between abdominal obese and non-abdominal obese subjects, and the associations of SOCS3 methylation levels with abdominal obesity were both positive and negative, which may be caused by the different status of DNA methylation at different sites in gene expression regulation. In general, the hyper-methylation level of CpG island cytosine in the gene promoter and its nearby region would silence gene expression, while the hypo-methylation level would reactivate the expression of silenced genes [32,33]. Meanwhile, hyper-methylation in the exon and intron regions (the gene body) may elevate gene expression [34,35,36].

The potential mechanisms responsible for the association of SOCS3 methylation levels with abdominal obesity may be elucidated by the following information. SOCS3, a key protein in many pathological events, such as diabetes and immune disorders, has emerged as the most essential regulator in the inhibition of the Janus kinase (JAK)-signal transducer and activator of transcription (STAT) pathway among the SOCS family [37,38,39]. Moreover, when the body is suffering from metabolic abnormalities, such as obesity, SOCS3 is involved in the inhibition of inflammatory cytokines and crucial hormones linked to energy metabolism, such as insulin signaling and leptin [40]. However, SOCS3 methylation results in the lower expression level of SOCS3 [38]. Therefore, the hyper-methylation of SOCS3 in the gene promoter and its nearby region silences the inhibitory effect on leptin and insulin of SOCS3, whereas hyper-methylation in the gene body facilitates it to inhibit the activity of insulin and leptin, resulting in a high risk of obesity. In addition, evidence has suggested that the increased SOCS3 expression regulates insulin signaling by inhibiting phosphorylation of the insulin receptor substrate (IRS) [41]. More epidemiological studies are needed to further investigate the mechanisms of different methylation sites with metabolic diseases (abdominal obesity, etc.) to provide more relevant evidence in the future.

Several studies have estimated the relationship between SOCS3 methylation and obesity; however, considering the cross-sectional nature of those traditional observational studies, they could not determine the causal effect between risk factors and disease. However, in the current study, based on the four SNPs (rs12953258, rs2280148, rs4969168 and rs4969170) of SOCS3 as instrumental variables, the Mendelian randomization analyses found a causal association between methylation levels of SOCS3 and abdominal obesity, which suggested that the SOCS3 gene might play a role in the pathogenesis of abdominal obesity and could be potentially used as a marker for attenuated or aggressive disease in the Asian population. Mendelian randomization is an approach to exploring the observational causal association of modifiable risk factors with health outcomes by using genetic variants [42], in which reverse causality might be avoided due to the characteristics of randomly distributed genetic variants at conception [43]. In this study, the causal association between the methylation levels of SOCS3 and abdominal obesity was established and some novelty sites for the association were found. However, more studies are needed to interpret the causal association, due to the limitation of instrumental variables in determining causal estimates.

Several limitations in the study should be noted. The temporality between DNA methylation levels and abdominal obesity could not be ascertained due to the case–control nature of the current study. In addition, the association between SOCS3 methylation level and abdominal obesity was found only in the Chinese rural population, so caution should be taken when generalizing the findings to other populations. It is necessary to explore the relationship between DNA methylation of SOCS3 and abdominal obesity in multicenter studies.

## 5. Conclusions

The current study provided evidence in support of the causal associations of the SOCS3 methylation level with abdominal obesity in the Chinese rural population, which suggested that SOCS3 methylation might be potentially used as a marker for development of abdominal obesity.

## Figures and Tables

**Table 1:** Distributions of characteristics of the participant by with and without abdominal obesity.

Variables	Total (n = 1064)	Abdominal Obesity (n = 471)	Non-Abdominal Obesity (n = 593)	*p*-Value
Age (years, mean ± SD)	59.52 ± 8.67	58.70 ± 8.46	60.17 ± 8.78	0.006
WC (cm, mean ± SD)	83.23 ± 10.78	91.82 ± 7.92	76.40 ± 7.32	<0.001
Gender, n (%)				<0.001
Men	459 (43.14)	138 (29.30)	321 (54.13)	
Women	605(56.86)	333 (70.70)	272 (45.87)	
Education levels, n (%)				0.843
Elementary school or below	586 (55.08)	261 (55.41)	325 (54.81)	
Junior high school or above	478 (44.92)	210 (44.59)	268 (45.19)	
Marital status, n (%)				0.819
Married/cohabiting	953 (89.57)	423 (89.81)	530 (89.38)	
Widowed/single/divorced/separation	111 (10.43)	48 (10.19)	63 (10.62)	
Average monthly income of family, n (%)				0.510
CNY < 500	426 (40.04)	194 (41.19)	232 (39.12)	
CNY 500 ~	336 (31.58)	140 (29.72)	196 (33.05)	
CNY ≥ 1000	302 (28.38)	137 (29.09)	165 (27.83)	
High-fat diet, n (%)				0.055
Yes	205 (19.27)	103 (21.87)	102 (17.20)	
No	859 (80.73)	368 (78.13)	491 (82.80)	
More vegetable and fruit intake, n (%)				0.482
Yes	690 (64.85)	300 (63.69)	390 (65.77)	
No	374 (35.15)	171 (36.31)	203 (34.23)	
Smoking status, n (%)				<0.001
Never	762 (71.62)	379 (80.47)	383 (64.59)	
Ever	88 (8.27)	39 (8.28)	49 (8.26)	
Current	214 (20.11)	53 (11.25)	161 (27.15)	
Drinking status, n (%)				0.063
Never	841 (79.04)	386 (81.95)	455 (76.73)	
Ever	62 (5.83)	20 (4.25)	42 (7.08)	
Current	161 (15.13)	65 (13.80)	96 (16.19)	
Physical activity (n, %)				0.001
Low	282 (26.50)	137 (29.09)	145 (24.45)	
Moderate	478 (44.92)	226 (47.98)	252 (42.50)	
High	304 (28.57)	108 (22.93)	196 (33.05)	
Family history of T2DM (n, %)				0.395
Yes	30 (2.82)	11 (2.34)	19 (3.20)	
No	1034 (97.18)	460 (97.66)	574 (96.80)	

Abbreviation: SD, standard deviation; WC, waist circumference; T2DM, type 2 diabetes.

**Table 2:** Associations of SOCS3 methylation level with abdominal obesity.

CpG Sites	Location	Distance2TSS	Abdominal Obesity Median (IQR)	Non-Abdominal Obesity Median (IQR)	*OR* (95% *CI*)	*p*-Value
Chr17:76356178	Promoter	−18	0.005 (0.003, 0.007)	0.005 (0.004, 0.007)	0.795 (0.679, 0.930)	0.004
Chr17:76356190	Promoter	−30	0.008 (0.006, 0.012)	0.009 (0.006, 0.014)	0.841 (0.721, 0.983)	0.029
Chr17:76356054	Exon	106	0.012 (0.009, 0.014)	0.012 (0.010, 0.015)	0.786 (0.672, 0.919)	0.003
Chr17:76356084	Exon	76	0.025 (0.020, 0.030)	0.026 (0.021, 0.031)	0.851 (0.732, 0.988)	0.034
Chr17:76356099	Exon	61	0.007 (0.005, 0.009)	0.007 (0.005, 0.010)	0.838 (0.719, 0.976)	0.023
Chr17:76354927	Exon	1233	0.338 (0.282, 0.405)	0.331 (0.269, 0.388)	1.280 (1.064, 1.539)	0.009
Chr17:76354934	Exon	1226	0.540 (0.476, 0.610)	0.531 (0.459, 0.592)	1.284 (1.067, 1.546)	0.008
Chr17:76354947	Exon	1213	0.454 (0.388, 0.535)	0.445 (0.373, 0.514)	1.272 (1.050, 1.541)	0.014
Chr17:76354955	Exon	1205	0.422 (0.354, 0.500)	0.410 (0.340, 0.483)	1.286 (1.064, 1.554)	0.009
Chr17:76354963	Exon	1197	0.283 (0.224, 0.345)	0.273 (0.217, 0.334)	1.277 (1.059, 1.539)	0.010
Chr17:76354965	Exon	1195	0.344 (0.286, 0.409)	0.332 (0.272, 0.395)	1.272 (1.056, 1.533)	0.011
Chr17:76354984	Exon	1176	0.287 (0.232, 0.361)	0.277 (0.223, 0.344)	1.266 (1.053, 1.522)	0.012
Chr17:76354990	Exon	1170	0.119 (0.088, 0.156)	0.113 (0.084, 0.147)	1.243 (1.044, 1.480)	0.015
Chr17:76355009	Exon	1151	0.287 (0.225, 0.354)	0.271 (0.214, 0.345)	1.282 (1.065, 1.543)	0.009
Chr17:76355014	Exon	1146	0.351 (0.284, 0.437)	0.341 (0.271, 0.412)	1.310 (1.084, 1.584)	0.005
Chr17:76355017	Exon	1143	0.277 (0.226, 0.341)	0.270 (0.214, 0.328)	1.277 (1.061, 1.537)	0.010
Chr17:76355020	Exon	1140	0.271 (0.216, 0.333)	0.260 (0.207, 0.321)	1.324 (1.102, 1.591)	0.003
Chr17:76355029	Exon	1131	0.274 (0.216, 0.334)	0.263 (0.207, 0.322)	1.266 (1.051, 1.525)	0.013
Chr17:76355044	Exon	1116	0.327 (0.258, 0.404)	0.316 (0.249, 0.388)	1.297 (1.073, 1.569)	0.007
Chr17:76355061	Exon	1099	0.410 (0.334, 0.493)	0.397 (0.323, 0.479)	1.325 (1.092, 1.608)	0.004
Chr17:76355068	Exon	1092	0.279 (0.213, 0.360)	0.268 (0.201, 0.342)	1.276 (1.056, 1.542)	0.012
Chr17:76355089	Exon	1071	0.293 (0.225, 0.375)	0.280 (0.210, 0.360)	1.285 (1.060, 1.557)	0.011
Chr17:76355115	Exon	1045	0.277 (0.210, 0.359)	0.269 (0.200, 0.344)	1.242 (1.026, 1.504)	0.026

Abbreviation: IQR, interquartile range; *OR*, odds ratio; *CI*, confidence interval. Adjusted for age, gender, education levels, marital status, average monthly income of family, high-fat diet, more vegetable and fruit intake, smoking status, drinking status, physical activity and family history of type 2 diabetes.

**Table 3:** Associations between SNP of SOCS3 and abdominal obesity.

SNP	Total	Abdominal Obesity	Non-Abdominal Obesity	*p*-Value	*OR* (95% *CI*)	*HWE p*-Value
rs12953258				0.540		0.235
GG	473 (44.79)	205 (43.80)	268 (45.58)		1.00	
GT	486 (46.02)	215 (45.94)	271 (46.09)		0.985 (0.750, 1.295)	
TT	97 (9.19)	48 (10.26)	49 (8.33)		1.111 (0.700, 1.764)	
Each T increase					1.027 (0.841, 1.255)	
rs2280148				0.392		0.967
GG	661 (62.59)	303 (64.88)	358 (60.78)		1.00	
GT	344 (32.58)	143 (30.62)	201 (34.13)		0.869 (0.656, 1.151)	
TT	51 (4.83)	21 (4.50)	30 (5.09)		0.851 (0.461, 1.571)	
Each T increase					0.892 (0.714, 1.115)	
rs4969168				0.208		0.584
GG	209 (19.75)	85 (18.12)	124 (21.05)		1.00	
GA	542 (51.23)	236 (50.32)	306 (51.95)		0.938 (0.663, 1.328)	
AA	307 (29.02)	148 (31.56)	159 (27.00)		1.145 (0.783, 1.675)	
Each A increase					1.087 (0.900, 1.311)	
rs4969170				0.931		0.999
GG	850 (80.26)	374 (79.74)	476 (80.68)		1.00	
GA	198 (18.70)	90 (19.19)	108 (18.30)		1.059 (0.759, 1.476)	
AA	11 (1.04)	5 (1.07)	6 (1.02)		1.374 (0.385, 4.908)	
Each A increase					1.080 (0.799, 1.461)	
rs9892622				0.258		0.828
GG	215 (20.30)	91 (19.40)	124 (21.02)		1.00	
GA	526 (49.67)	225 (47.97)	301 (51.02)		0.950 (0.674, 1.338)	
AA	318 (30.03)	153 (32.63)	165 (27.96)		1.203 (0.829, 1.746)	
Each A increase					1.113 (0.926, 1.339)	
rs9914220				0.016		0.567
CC	390 (36.83)	194 (41.36)	196 (33.22)		1.00	
CT	479 (45.23)	202 (43.07)	277 (46.95)		0.753 (0.565, 1.003)	
TT	190 (17.94)	73 (15.57)	117 (19.83)		0.705 (0.484, 1.027)	
Each T increase					0.823 (0.686, 0.988)	

Abbreviation: *OR*, odds ratio; *CI*, confidence interval. Adjusted for age, gender, education levels, marital status, average monthly income of family, high-fat diet, more vegetable and fruit intake, smoking status, drinking status, physical activity and family history of type 2 diabetes. HWE: Hardy–Weinberg equilibrium.

**Table 4:** Mendelian randomization causal-effect estimates for SOCS3 methylation level with abdominal obesity.

Method	*β* (95%*CI*)	*p*-Value
Maximum-likelihood method	5.342 (0.215, 10.469)	0.041
MR-IVW	4.911 (0.259, 9.564)	0.039
MR-median	5.117 (−1.356, 11.590)	0.121

Abbreviation: *β*, correlation coefficient; *CI*, confidence interval; MR-IVW, Mendelian randomization method of penalized inverse variance weighted; MR-median, Mendelian randomization method of median.

## Data Availability

The original contributions presented in the study are included in the article/Supplementary Material; further inquiries can be directed to the corresponding authors.

## Supplementary Materials

The following supporting information can be downloaded at: https://www.mdpi.com/article/10.3390/nu14183824/s1, Table S1: Relationship between SOCS3 methylation level of and abdominal obesity; Table S2: *p* values for the overall and the non-linear association test for SOCS3 methylation level with abdominal obesity in the restricted cubic spline; Table S3: Relationship between SNP and methylation level of SOCS3; Table S4: Relationship between SNP of SOCS3 and abdominal obesity; Table S5: Effects of each CpG site at each SNP of SOCS3 on abdominal obesity using the Wald ratio method; Figure S1: The dose–response relationship between methylation levels of SOCS3 with obesity.
