# Supplementary material for: Mendelian Randomization Analysis of the Association of SOCS3 Methylation with Abdominal Obesity
Source: Nutrients. 2022 Sep 16;14(18):3824. doi: 10.3390/nu14183824 (PMC9503364; doi:10.3390/nu14183824)
Supplement: Supplementary file 1 [file nutrients-14-03824-s001.zip › nutrients-1865522-supplementary.pdf]

Supplementary Table S1. Relationship between SOCS3 methylation level of and abdominal obesity [a]

CpG sites	Location	Distance to TSS	Abdominal obesity Median (IQR)	Non-abdominal obesity Median (IQR)	*OR* (95% *CI*)	*P*-value
Chr17:76355136	exon	1024	0.079 (0.061, 0.103)	0.080 (0.061, 0.102)	1.054 (0.911, 1.220)	0.479
Chr17:76355146	exon	1014	0.019 (0.014, 0.024)	0.018 (0.013, 0.023)	1.077 (0.929, 1.248)	0.327
Chr17:76355149	exon	1011	0.022 (0.017, 0.029)	0.022 (0.016, 0.028)	1.032 (0.911, 1.170)	0.617
Chr17:76355152	exon	1008	0.032 (0.024, 0.043)	0.031 (0.023, 0.041)	1.117 (0.956, 1.305)	0.165
Chr17:76355179	exon	981	0.050 (0.039, 0.068)	0.049 (0.037, 0.065)	1.162 (0.988, 1.366)	0.069
Chr17:76355183	exon	977	0.013 (0.009, 0.018)	0.013 (0.009, 0.017)	1.028 (0.879, 1.202)	0.731
Chr17:76355192	exon	968	0.010 (0.006, 0.014)	0.010 (0.007, 0.014)	1.034 (0.877, 1.221)	0.689
Chr17:76355202	exon	958	0.011 (0.007, 0.015)	0.011 (0.008, 0.015)	0.981 (0.831, 1.158)	0.820
Chr17:76355204	exon	956	0.009 (0.006, 0.012)	0.009 (0.006, 0.012)	1.029 (0.876, 1.209)	0.729
Chr17:76355207	exon	953	0.011 (0.007, 0.015)	0.010 (0.007, 0.014)	1.140 (0.982, 1.324)	0.084
Chr17:76355210	exon	950	0.011 (0.007, 0.016)	0.011 (0.007, 0.015)	1.148 (0.982, 1.343)	0.083
Chr17:76355225	exon	935	0.010 (0.007, 0.014)	0.010 (0.007, 0.014)	1.078 (0.915, 1.270)	0.368
Chr17:76355227	exon	933	0.008 (0.005, 0.012)	0.008 (0.006, 0.012)	1.029 (0.890, 1.190)	0.699
Chr17:76355234	exon	926	0.014 (0.011, 0.020)	0.014 (0.011, 0.018)	1.095 (0.941, 1.275)	0.240
Chr17:76355241	exon	919	0.007 (0.004, 0.010)	0.008 (0.005, 0.011)	0.847 (0.717, 1.000)	0.049
Chr17:76355245	exon	915	0.014 (0.009, 0.018)	0.013 (0.010, 0.017)	1.070 (0.907, 1.262)	0.422
Chr17:76355251	exon	909	0.010 (0.007, 0.014)	0.010 (0.007, 0.014)	1.048 (0.889, 1.235)	0.578
Chr17:76355258	promoter	902	0.012 (0.008, 0.016)	0.013 (0.009, 0.016)	0.933 (0.787, 1.106)	0.424
Chr17:76355268	promoter	892	0.014 (0.011, 0.019)	0.015 (0.011, 0.020)	0.876 (0.744, 1.032)	0.113
Chr17:76355280	promoter	880	0.015 (0.011, 0.020)	0.014 (0.010, 0.019)	1.070 (0.910, 1.258)	0.413
Chr17:76355282	promoter	878	0.010 (0.007, 0.014)	0.010 (0.007, 0.014)	0.963 (0.845, 1.098)	0.576
Chr17:76355286	promoter	874	0.011 (0.008, 0.016)	0.011 (0.008, 0.015)	1.001 (0.839, 1.195)	0.989
Chr17:76355288	promoter	872	0.009 (0.007, 0.013)	0.010 (0.007, 0.013)	1.065 (0.918, 1.237)	0.406
Chr17:76355299	promoter	861	0.010 (0.007, 0.014)	0.011 (0.007, 0.014)	0.984 (0.845, 1.146)	0.837
Chr17:76355312	promoter	848	0.008 (0.005, 0.011)	0.008 (0.005, 0.011)	1.073 (0.922, 1.249)	0.362
Chr17:76355314	promoter	846	0.009 (0.005, 0.013)	0.008 (0.006, 0.011)	1.169 (0.985, 1.387)	0.074
Chr17:76355318	promoter	842	0.009 (0.006, 0.012)	0.008 (0.006, 0.011)	1.032 (0.877, 1.215)	0.702
Chr17:76355323	promoter	837	0.009 (0.006, 0.013)	0.010 (0.007, 0.013)	1.051 (0.936, 1.179)	0.402
Chr17:76355328	promoter	832	0.007 (0.004, 0.010)	0.007 (0.005, 0.010)	0.949 (0.803, 1.122)	0.541
Chr17:76355343	promoter	817	0.009 (0.006, 0.012)	0.008 (0.006, 0.012)	0.967 (0.839, 1.114)	0.642
Chr17:76355346	promoter	814	0.009 (0.006, 0.012)	0.009 (0.006, 0.012)	0.988 (0.844, 1.157)	0.879
Chr17:76355350	promoter	810	0.006 (0.004, 0.008)	0.006 (0.004, 0.009)	0.937 (0.798, 1.100)	0.427
Chr17:76356056	promoter	104	0.007 (0.005, 0.009)	0.007 (0.005, 0.010)	0.885 (0.761, 1.029)	0.113
Chr17:76356067	promoter	93	0.016 (0.013, 0.019)	0.016 (0.014, 0.020)	0.933 (0.820, 1.061)	0.291
Chr17:76356069	promoter	91	0.011 (0.008, 0.013)	0.011 (0.008, 0.013)	1.007 (0.873, 1.161)	0.926
Chr17:76356072	promoter	88	0.007 (0.006, 0.009)	0.008 (0.006, 0.010)	0.927 (0.816, 1.052)	0.239
Chr17:76356088	promoter	72	0.008 (0.006, 0.010)	0.008 (0.006, 0.010)	1.019 (0.887, 1.171)	0.786
Chr17:76356092	promoter	68	0.008 (0.006, 0.010)	0.008 (0.006, 0.010)	0.954 (0.827, 1.100)	0.515
Chr17:76356109	promoter	51	0.007 (0.005, 0.009)	0.007 (0.005, 0.009)	0.944 (0.816, 1.093)	0.441
Chr17:76356113	promoter	47	0.008 (0.006, 0.010)	0.008 (0.006, 0.010)	0.938 (0.800, 1.100)	0.432
Chr17:76356119	promoter	41	0.010 (0.007, 0.012)	0.010 (0.007, 0.013)	0.925 (0.790, 1.083)	0.334
Chr17:76356124	promoter	36	0.008 (0.006, 0.010)	0.008 (0.006, 0.010)	0.933 (0.805, 1.080)	0.351
Chr17:76356133	promoter	27	0.007 (0.005, 0.009)	0.007 (0.005, 0.009)	0.953 (0.822, 1.105)	0.526
Chr17:76356152	promoter	8	0.006 (0.005, 0.008)	0.006 (0.005, 0.008)	0.948 (0.831, 1.081)	0.426
Chr17:76356158	promoter	2	0.010 (0.007, 0.012)	0.010 (0.008, 0.012)	0.958 (0.832, 1.103)	0.551

Chr17:76356161	promoter	-1	0.011 (0.008, 0.013)	0.011 (0.009, 0.013)	0.924 (0.801, 1.065)	0.273
Chr17:76356163	promoter	-3	0.006 (0.005, 0.009)	0.007 (0.005, 0.008)	0.923 (0.804, 1.059)	0.252
Chr17:76356170	promoter	-10	0.010 (0.007, 0.012)	0.010 (0.007, 0.012)	0.979 (0.861, 1.114)	0.751
Chr17:76356172	promoter	-12	0.007 (0.005, 0.009)	0.007 (0.005, 0.009)	0.937 (0.807, 1.089)	0.398
Chr17:76356176	promoter	-16	0.007 (0.006, 0.009)	0.008 (0.006, 0.010)	1.004 (0.897, 1.124)	0.944
Chr17:76356194	promoter	-34	0.012 (0.008, 0.014)	0.011 (0.009, 0.014)	0.910 (0.782, 1.058)	0.219
Chr17:76356197	promoter	-37	0.011 (0.009, 0.014)	0.011 (0.009, 0.014)	0.961 (0.843, 1.095)	0.547
Chr17:76356199	promoter	-39	0.009 (0.006, 0.011)	0.009 (0.007, 0.012)	1.016 (0.955, 1.081)	0.606
Chr17:76356204	promoter	-44	0.008 (0.006, 0.010)	0.008 (0.006, 0.010)	0.942 (0.798, 1.113)	0.482
Chr17:76356211	promoter	-51	0.008 (0.005, 0.010)	0.008 (0.006, 0.009)	0.978 (0.872, 1.098)	0.708
Chr17:76356226	promoter	-66	0.008 (0.006, 0.010)	0.008 (0.006, 0.010)	0.997 (0.873, 1.138)	0.959
Chr17:76356228	promoter	-68	0.007 (0.005, 0.009)	0.007 (0.006, 0.009)	1.032 (0.935, 1.139)	0.530
Chr17:76356232	promoter	-72	0.008 (0.006, 0.010)	0.008 (0.006, 0.010)	0.896 (0.762, 1.052)	0.180
Chr17:76354981	exon	1179	0.141 (0.110, 0.180)	0.139 (0.108, 0.173)	1.191 (0.992, 1.431)	0.062
Chr17:76354582	exon	1578	0.785 (0.749, 0.819)	0.788 (0.745, 0.819)	1.041 (0.881, 1.231)	0.636
Chr17:76354588	exon	1572	0.872 (0.844, 0.897)	0.871 (0.848, 0.901)	0.949 (0.81, 1.112)	0.516
Chr17:76354596	exon	1564	0.924 (0.900, 0.948)	0.927 (0.909, 0.946)	0.896 (0.76, 1.058)	0.196
Chr17:76354621	exon	1539	0.456 (0.413, 0.503)	0.453 (0.413, 0.500)	1.050 (0.887, 1.244)	0.571
Chr17:76354638	exon	1522	0.864 (0.830, 0.892)	0.864 (0.828, 0.891)	1.070 (0.904, 1.266)	0.433
Chr17:76354663	exon	1497	0.856 (0.822, 0.885)	0.858 (0.828, 0.888)	0.967 (0.816, 1.146)	0.697
Chr17:76354669	exon	1491	0.750 (0.719, 0.790)	0.758 (0.721, 0.795)	1.006 (0.854, 1.185)	0.945
Chr17:76354724	exon	1436	0.688 (0.649, 0.729)	0.693 (0.647, 0.734)	1.010 (0.863, 1.182)	0.903
Chr17:76354732	exon	1428	0.80 (0.764, 0.839)	0.811 (0.774, 0.846)	0.973 (0.819, 1.156)	0.758
Chr17:76354741	exon	1419	0.713 (0.666, 0.767)	0.721 (0.664, 0.769)	1.123 (0.946, 1.333)	0.186
Chr17:76354763	exon	1397	0.731 (0.684, 0.779)	0.736 (0.689, 0.778)	1.020 (0.859, 1.212)	0.820

Abbreviation: IQR, interquartile range; OR, odds ratio; CI, confidence interval.

Distance to TSS: The relative distance (in bp) to transcriptional start site (TSS), the minus sign indicates that the site is at the upstream of TSS. [a] Adjusted for age, gender, education levels, marital status, average monthly income of family, high-fat diet, more vegetables and fruits intake, smoking status, drinking status, physical activity, family history of type 2 diabetes.

Supplementary Table S2. *P* values for the overall and the non-linear association test for SOCS3 methylation level with abdominal obesity in the restricted cubic spline [a]

CpG sites	χ^2	*P* for the overall association test	χ^2	*P* for the non-linear association test
Chr17:76356054	12.47	0.002	5.04	0.025
Chr17:76356084	4.31	0.116	0.19	0.664
Chr17:76356099	4.90	0.086	0.37	0.544
Chr17:76356178	7.94	0.019	0.02	0.900
Chr17:76356190	7.86	0.020	0.35	0.555
Chr17:76354927	6.50	0.039	0.14	0.707
Chr17:76354934	6.95	0.031	0.38	0.538
Chr17:76354947	5.92	0.052	0.13	0.719
Chr17:76354955	6.32	0.043	0.05	0.819
Chr17:76354963	6.78	0.034	0.44	0.507
Chr17:76354965	6.16	0.046	0.10	0.754
Chr17:76354984	6.03	0.049	0.01	0.930
Chr17:76354990	5.98	0.050	0.25	0.620
Chr17:76355009	6.75	0.034	0.10	0.752
Chr17:76355014	7.67	0.022	0.24	0.628
Chr17:76355017	7.12	0.028	0.74	0.390
Chr17:76355020	9.33	0.009	0.75	0.387
Chr17:76355029	6.17	0.046	0.31	0.578
Chr17:76355044	7.21	0.027	0.30	0.582
Chr17:76355061	8.56	0.014	0.60	0.438
Chr17:76355068	6.41	0.041	0.23	0.634
Chr17:76355089	6.22	0.045	0.14	0.709
Chr17:76355115	4.44	0.109	<0.01	0.990

[a] Adjusted for age, gender, education levels, marital status, average monthly income of family, high-fat diet, more vegetables and fruits intake, smoking status, drinking status, physical activity, family history of type 2 diabetes.

Supplementary Table S3. Relationship between SNP and methylation level of SOCS3

SNPs	CpGs	*β*	SE	*P*
rs12953258				
	Chr17:76356084	-0.0041	0.0004	<0.0001
	Chr17:76356099	-0.0006	0.0002	0.0005
rs2280148				
	Chr17:76354963	-0.0098	0.0044	0.0262
	Chr17:76354984	-0.0109	0.0047	0.0211
	Chr17:76354990	-0.0054	0.0026	0.0366
	Chr17:76355044	-0.0104	0.0052	0.0466
	Chr17:76355061	-0.0116	0.0058	0.0454
	Chr17:76355068	-0.0112	0.0053	0.0339
	Chr17:76355089	-0.0107	0.0054	0.0504
	Chr17:76355115	-0.0105	0.0053	0.0491
	Chr17:76356084	0.0020	0.0004	<0.0001
rs4969168				
	Chr17:76356084	-0.0023	0.0004	<0.0001
rs4969170				
	Chr17:76354927	0.0133	0.0061	0.0303
	Chr17:76354955	0.0143	0.0071	0.0454
	Chr17:76354963	0.0131	0.0059	0.0273
	Chr17:76354965	0.0127	0.0062	0.0415
	Chr17:76354990	0.0079	0.0035	0.0228
	Chr17:76355009	0.0145	0.0066	0.0297
	Chr17:76355014	0.0152	0.0072	0.0356
	Chr17:76355017	0.0135	0.0059	0.0213
	Chr17:76355029	0.0123	0.0060	0.0394
	Chr17:76355044	0.0154	0.0070	0.0295
	Chr17:76355068	0.0146	0.0071	0.0409
	Chr17:76355089	0.0170	0.0073	0.0205
	Chr17:76355115	0.0170	0.0072	0.0180
rs9914220				
	Chr17:76356084	0.0008	0.0003	0.0247
	Chr17:76354927	-0.0104	0.0037	0.0050
	Chr17:76354934	-0.0115	0.0041	0.0056
	Chr17:76354947	-0.0134	0.0043	0.0021
	Chr17:76354955	-0.012	0.0043	0.0051
	Chr17:76354963	-0.0105	0.0036	0.0035
	Chr17:76354965	-0.0097	0.0037	0.0096
	Chr17:76354984	-0.0109	0.0039	0.0047
	Chr17:76354990	-0.0058	0.0021	0.0050
	Chr17:76355009	-0.0103	0.0040	0.0103
	Chr17:76355014	-0.0102	0.0043	0.0189
	Chr17:76355017	-0.0094	0.0035	0.0078
	Chr17:76355020	-0.0078	0.0036	0.0280

	Chr17:76355029	-0.0089	0.0036	0.0135
	Chr17:76355044	-0.0114	0.0042	0.0072
	Chr17:76355061	-0.0123	0.0047	0.0092
	Chr17:76355068	-0.0110	0.0043	0.0106
	Chr17:76355089	-0.0117	0.0044	0.0080
	Chr17:76355115	-0.0115	0.0043	0.0076

Abbreviation: β, correlation coefficient; SE, standard error.

Supplementary Table S4. Relationship between SNP of SOCS3 and abdominal obesity [a]

**SNPs**	***β***	**SE**	***P***
rs12953258	0.027	0.102	0.793
rs2280148	-0.114	0.114	0.317
rs4969168	0.083	0.096	0.387
rs4969170	0.077	0.154	0.615

a Adjusted for age, gender, education levels, marital status, average monthly income of family, high-fat diet, more vegetables and fruits intake, smoking status, drinking status, physical activity, family history of type 2 diabetes.

Supplementary Table S5. Effects of each CpG site at each SNP of SOCS3 on abdominal obesity by Wald ratio method

CpGs	SNPs	Wald ratio	SE
Chr17:76356084	rs12953258	-6.585	24.886
Chr17:76356099	rs12953258	-45.000	170.66
Chr17:76354963	rs2280148	11.633	12.751
Chr17:76354984	rs2280148	10.459	11.390
Chr17:76354990	rs2280148	21.111	23.431
Chr17:76355044	rs2280148	10.962	12.255
Chr17:76355061	rs2280148	9.828	10.988
Chr17:76355068	rs2280148	10.179	11.261
Chr17:76355089	rs2280148	10.654	11.934
Chr17:76355115	rs2280148	10.857	12.162
Chr17:76356084	rs2280148	-57.000	58.129
Chr17:76356084	rs4969168	-36.087	42.208
Chr17:76354927	rs4969170	5.789	11.880
Chr17:76354955	rs4969170	5.385	11.096
Chr17:76354963	rs4969170	5.878	12.050
Chr17:76354965	rs4969170	6.063	12.482
Chr17:76354990	rs4969170	9.747	19.966
Chr17:76355009	rs4969170	5.310	10.892
Chr17:76355014	rs4969170	5.066	10.412
Chr17:76355017	rs4969170	5.704	11.677
Chr17:76355029	rs4969170	6.260	12.887
Chr17:76355044	rs4969170	5.000	10.255
Chr17:76355068	rs4969170	5.274	10.855
Chr17:76355089	rs4969170	4.529	9.265
Chr17:76355115	rs4969170	4.529	9.260

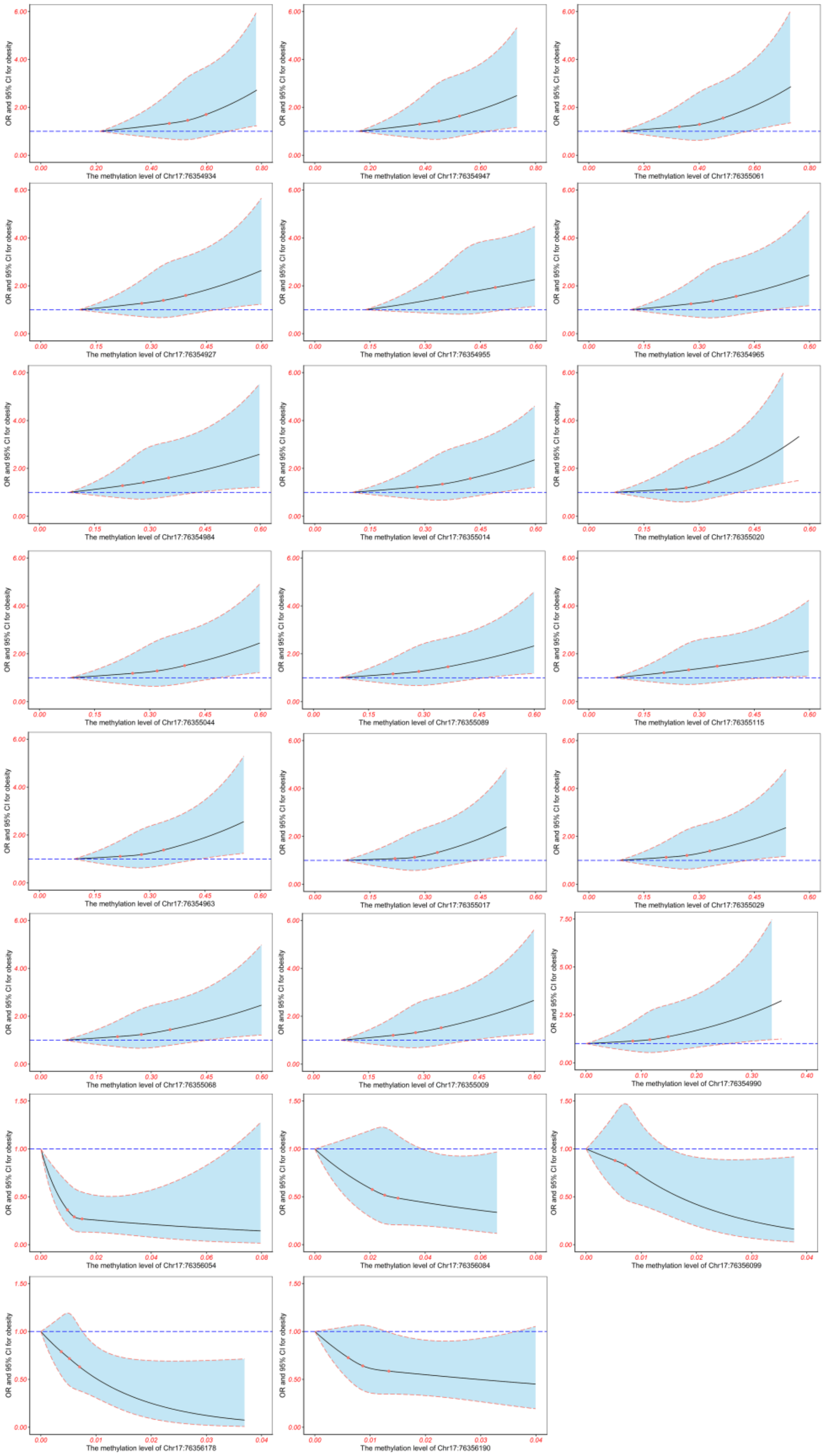

Supplementary Figure S1. The dose-response relationship between methylation level of SOCS3 with obesity (solid lines, *OR*s; dashed lines, 95% *CI*s). Adjusted for age, gender, education levels, marital status, average monthly income of family, high-fat diet, more vegetables and fruits intake, smoking status, drinking status, physical activity, family history of type 2 diabetes.
